# Lineage-specific defence systems of pandemic *Vibrio cholerae*

**DOI:** 10.1098/rstb.2024.0076

**Published:** 2025-09-04

**Authors:** Melanie Blokesch, Kimberley D. Seed

**Affiliations:** ^1^ Laboratory of Molecular Microbiology, Global Health Institute, School of Life Sciences, Ecole Polytechnique Federale de Lausanne (EPFL), Lausanne, Switzerland; ^2^ Department of Plant and Microbial Biology, University of California Berkeley, Berkeley, CA, USA

**Keywords:** bacteriophage, cholera, phage defence

## Abstract

Cholera remains a significant global health burden. The causative agent responsible for the ongoing cholera pandemic, which began in 1961, is the seventh pandemic El Tor (7PET) lineage of *Vibrio cholerae*. Over the past century, lineages of *V. cholerae* have been traced using phage typing schemes, DNA hybridization on microarrays and, more recently, comparative genomics enabled by next-generation sequencing. Such lineage tracing has provided essential insights into cholera transmission dynamics. Beyond their use as tools in typing schemes, phages have long been recognized as major players in cholera epidemiology. Importantly, the integration of comparative genomics, epidemiology and molecular studies has recently provided compelling evidence that bacterial defence systems, along with the evolutionary adaptations of phages to counteract them, play critical roles in the ongoing arms race between pandemic *V. cholerae* and their phages, with phage resistance likely influencing cholera epidemiology. In this review, we explore abundant and sporadic defence systems in sub-lineages of 7PET *V. cholerae* and describe how they protect their bacterial hosts from predatory phages. Additionally, we contrast these findings with the defence activities observed in the sixth pandemic classical lineage of *V. cholerae*. Finally, we discuss the experimental challenges and limitations associated with studying defence systems in *V. cholerae* and propose future directions to advance research in this field.

This article is part of the discussion meeting issue ‘The ecology and evolution of bacterial immune systems’.

## 1. Introduction

Cholera has been a global health concern for centuries. The ongoing seventh pandemic began in 1961 in Indonesia and has since spread worldwide, primarily originating from cholera-endemic regions in Asia, such as the Bay of Bengal. The causative agent is the bacterium *Vibrio cholerae*, with the seventh pandemic El Tor (7PET) lineage specifically responsible for the current pandemic.

Comparative genomic analyses of isolates collected at the onset and throughout the pandemic have provided valuable insights into the relatedness of these strains and their transmission dynamics [1–3]. These studies revealed that *V. cholerae* spread from the Bay of Bengal to other parts of the world through three major transmission waves [2]. Wave 1, which occurred between 1977 and 1992, spread globally and included the introduction of 7PET *V. cholerae* to South America via West Africa, forming what is now known as the West African–South American (WASA) lineage [2,4,5]. Subsequently, wave 1 strains were replaced by the more geographically restricted wave 2 and wave 3 lineages, which continue to cause cholera outbreaks today, particularly in South Asia and Africa [2,4,5]. The most recent, third transmission wave also led to the introduction of *V. cholerae* into Haiti and Yemen. These outbreaks were predominantly initiated by single introduction events [5–8], although some countries, such as Lebanon, have recently experienced two-strain cholera outbreaks [9]. Comparative genomic studies have thus been instrumental in identifying these transmission events.

**Figure 1.**
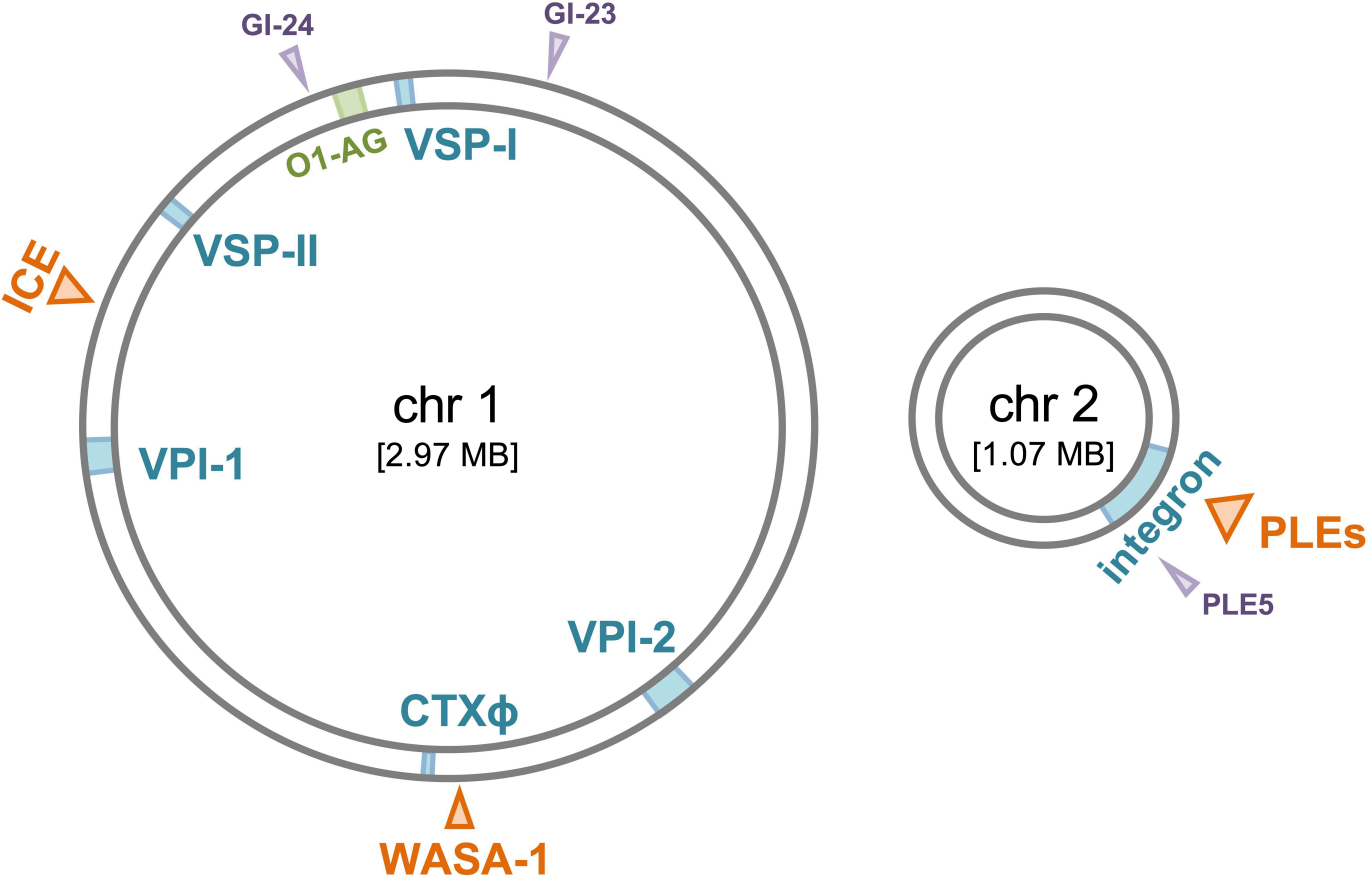
Mobile genetic elements (MGEs) in pandemic *V. cholerae*. Schematic representation of the two chromosomes of *V. cholerae*, showing the location and insertion sites of MGEs. Core pandemic MGEs, including CTXɸ, VPI-1, VPI-2, VSP-I, VSP-II and the integron are highlighted in blue, while variable MGEs are shown in orange. MGEs specific to the classical lineage are marked in purple. The O1 antigen-encoding region, which is common to all pandemic *V. cholerae* strains, is shown in green. This map is based on a resequenced and de novo assembled genome [15] of the reference strain N16961 [11].

Initial comparative genomic approaches using DNA hybridization on microarrays allowed researchers to identify only conserved versus lost genes [10] relative to the reference genome of the 7PET strain N16961, isolated in Bangladesh in 1975, on which the microarrays were based [11]. Despite their limitations, these early studies by Dziejman *et al.* were crucial for confirming the (almost) exclusive presence of the two known *Vibrio* pathogenicity islands 1 and 2 (VPI-1/2) in sixth pandemic classical and 7PET strains of pathogenic *V. cholerae* [10,12–14] (figure 1). They also confirmed the presence of SXT-like integrative and conjugative elements (ICE) starting with wave 2 strains [2,16], which were first described in the O139 serogroup-converted 7PET sub-lineage [17], and identified the 7PET-specific *Vibrio* seventh pandemic islands I and II (VSP-I/II) [10] (figure 1).

To categorize distinct phyletic lineages and reconstruct transmission events, next-generation sequencing-based comparative genomic studies initially excluded mobile genetic elements (MGEs) from phylogenetic analysis, instead focusing on single-nucleotide polymorphisms within the core genome [2]. However, while not always the primary focus, several of these studies also reported novel MGEs. For example, Chun *et al.* identified 73 genomic islands, each containing at least five open reading frames (ORFs), in a set of 23 *V*. *cholerae* strains, with only a minority of these MGEs present in all 7PET strains [1].

Mutreja *et al.* expanded the list of MGEs that are not universally present among all 7PET strains. This included the identification of a novel genomic island named WASA-1, which was found in an Angolan *V. cholerae* isolate as well as in all South American strains analysed in their study, making it a hallmark of the wave 1 WASA lineage [2] (figure 1). BLAST searches revealed that most genes carried by WASA-1 encode putative uncharacterized proteins, with a few candidates resembling phage proteins [2]. Further genomic analysis confirmed that WASA-1 is likely a prophage, homologous to prophages found in two other *Vibrio* species [18]. However, any encoded functions remained unknown until recently [19].

Collectively, while comparative genomic analyses allowed us to identify new MGEs and reconstruct transmission events, significant knowledge gaps remain in understanding why the introduction of *V. cholerae* leads to explosive outbreaks in some regions while becoming endemic or persisting at lower levels in others. It is tempting to speculate that the local composition of bacterial predators, including bacteriophages, and the defence systems of 7PET strains against them play a critical role in shaping the outbreak dynamics of these strains.

Defence systems are often clustered on MGEs, and recent research has significantly expanded the list of known phage defence systems that differ from classical restriction-modification and CRISPR systems [20–25]. This review highlights accessory MGEs of sixth and seventh pandemic *V. cholerae* that have been identified to exert phage defence functions.

The advantage of studying such accessory MGEs for their anti-phage activity is that well-characterized vibriophages isolated on pandemic *V. cholerae* strains are often susceptible to these defence systems. This allows for studies within the defence system’s native host and at native expression levels, as primarily described throughout this review. Combined with time series of coevolving bacteria-phage isolates, these studies provide valuable insights into the ecology and evolution of the defence systems and their bacterial hosts. Importantly, coevolving phages are expected to encode anti-defence strategies for defences common to their bacterial hosts (e.g. the entire 7PET lineage). As a result, studying common defence systems often requires heterologous hosts and access to respective phage collections. An accompanying article by Blokesch focuses on anti-phage/anti-plasmid systems common to 7PET strains, specifically those encoded on the core genomic islands VPI-2, VSP-I and VSP-II (figure 1) [26].

## 2. Vibriophages targeting *Vibrio cholerae*

### (a) Phage typing of *Vibrio cholerae*

To date, only a limited number of diverse *V. cholerae* phages have been described compared to the broader repertoire of vibriophages studied through longitudinal sampling and phylogenetic analysis in other species of the genus *Vibrio* [27,28].

Initially, *V. cholerae* phages were used for strain typing. For example, while primarily focusing on the strains’ lysotypes, Nicolle *et al.* also utilized a small set of phages to type strains [29]. Lysotypes were determined through the release of prophages following UV treatment or the application of diluted bleach. This approach facilitated the classification of strains, primarily distinguishing classical from 7PET isolates, as well as pandemic precursor strains [29]. Shortly afterwards, Basu & Mukerjee developed a phage typing scheme that classified *V. cholerae* strains into six major subcategories based on the use of five phages [30]. This study also provided valuable insights into the initial transmission events of the seventh pandemic, which officially began in 1961 in Celebes, Indonesia. Specifically, it revealed that only strains from two of the six subcategories observed in Celebes reached Pakistan and India between 1963 and 1964 [30]. This typing scheme was later extended to create a method better suited for typing all 7PET strains. This was achieved by including an additional five phages compared to the original work by Basu & Mukerjee [31].

Phage typing schemes were initially developed to differentiate strains and track transmission routes, much like comparative genomics would later achieve. However, the reasons behind the varying phage susceptibility patterns among different strains were not an active area of research at the time. Today, we understand that these phage typing schemes likely reflected differences in anti-phage defence systems between classical and 7PET strains, as well as among sub-lineages of 7PET strains, which is the focus of this review.

### (b) Role of phages in the epidemiology of *Vibrio cholerae*

Apart from the toxigenic conversion of ancestral strains by the cholera toxin-encoding lysogenic CTXɸ phage, which encodes the major virulence factor of pandemic *V. cholerae*, cholera toxin [32] (for more details on CTXɸ, see the accompanying article by Blokesch [26]), the role of lytic phages in the evolution and epidemiology of the pathogen has long been hypothesized [33–35]. For instance, a study conducted in Bangladesh reported an inverse correlation between peaks of cholera cases and the presence of certain lytic phages in environmental waters. Based on these findings, Faruque *et al.* proposed that phages play a key role in terminating cholera outbreaks and suggested that their depletion from the environment due to severe flooding, for example, might contribute to *V. cholerae* transmission [36]. A model was therefore proposed in which the proliferation of *V. cholerae* during an outbreak drives a subsequent increase in lytic phages. These phages are co-ingested by humans along with the pathogen, leading to their amplification and excretion in patients’ stool, which may ultimately contribute to the decline of the outbreak in cholera-endemic areas [36–38]. Faruque *et al.* further suggested that permissive bacterial hosts might contribute to higher concentrations of certain phages in the aquatic environment, potentially rendering the water unsuitable for the propagation of O1 pandemic *V. cholerae* [36].

The host strain’s ability to evade phage predation is thus a critical factor in the evolutionary arms race. While early studies attributed phage resistance primarily to altered phage receptors, such as flagella, pili or the O-antigen, these structures are often crucial for virulence and less likely to undergo alteration in a disease context [39,40]. Instead, we now understand that intracellular mechanisms of phage defence encoded by MGEs confer phage resistance while preserving the pathogenic potential of *V. cholerae*.

### (c) Well-characterized *Vibrio cholerae* phage isolates

The initial phage typing studies did not emphasize an in-depth understanding of the phages themselves, instead phenotyping them only at basic levels (e.g. through genomic restriction profiling and morphologies observed via transmission electron microscopy [31,41]). More recent research has sampled vibriophages from both the environment and cholera patient stool samples. For instance, Faruque and colleagues described a set of 27 phages (JSF1 through JSF27) isolated from surface water and cholera patients in Bangladesh [36,42]. However, subsequent comparative genomic analyses based on whole-genome sequences revealed a high degree of relatedness among these isolates [43]. Seed *et al*. focused on phage lineages frequently found in cholera stool, naming them International Centre for Diarrhoeal Disease Research, Bangladesh cholera phage 1 (ICP1; showing identity/similarity with JSF1, JSF4, and JSF5 [35,36]), ICP2 and ICP3 [44]. Among these three, ICP1 (a myovirus, approx. 125 kb genome) emerged as the dominant lytic phage, whereas ICP2 and ICP3 (podoviruses, approx. 50 and 40 kb genomes, respectively) exhibited more transient appearances [44]. Longitudinal studies combined with comparative genomics have enabled researchers to trace the evolutionary trajectory of these phages, with a specific focus on ICP1, over time [45]. Moreover, a recent study utilized metagenomics to analyse both *V. cholerae* and ICP1 in cholera patients in Bangladesh in a year-long, nationwide study, establishing correlations between the ICP1-to-*V. cholerae* ratio and the severity of dehydration [46]. Though it is not clear if ICP1 is the dominant phage associated with *V. cholerae* outside of Bangladesh, ICP1 has been detected in India, the Democratic Republic of the Congo, South Sudan and Yemen [8,45]. Collectively, these well-characterized ICP phages have served as essential tools for uncovering the variable phage defence systems present in pandemic *V. cholerae*, as outlined below.

## 3. Phage-inducible chromosomal island-like elements (PLEs)

**Figure 2.**
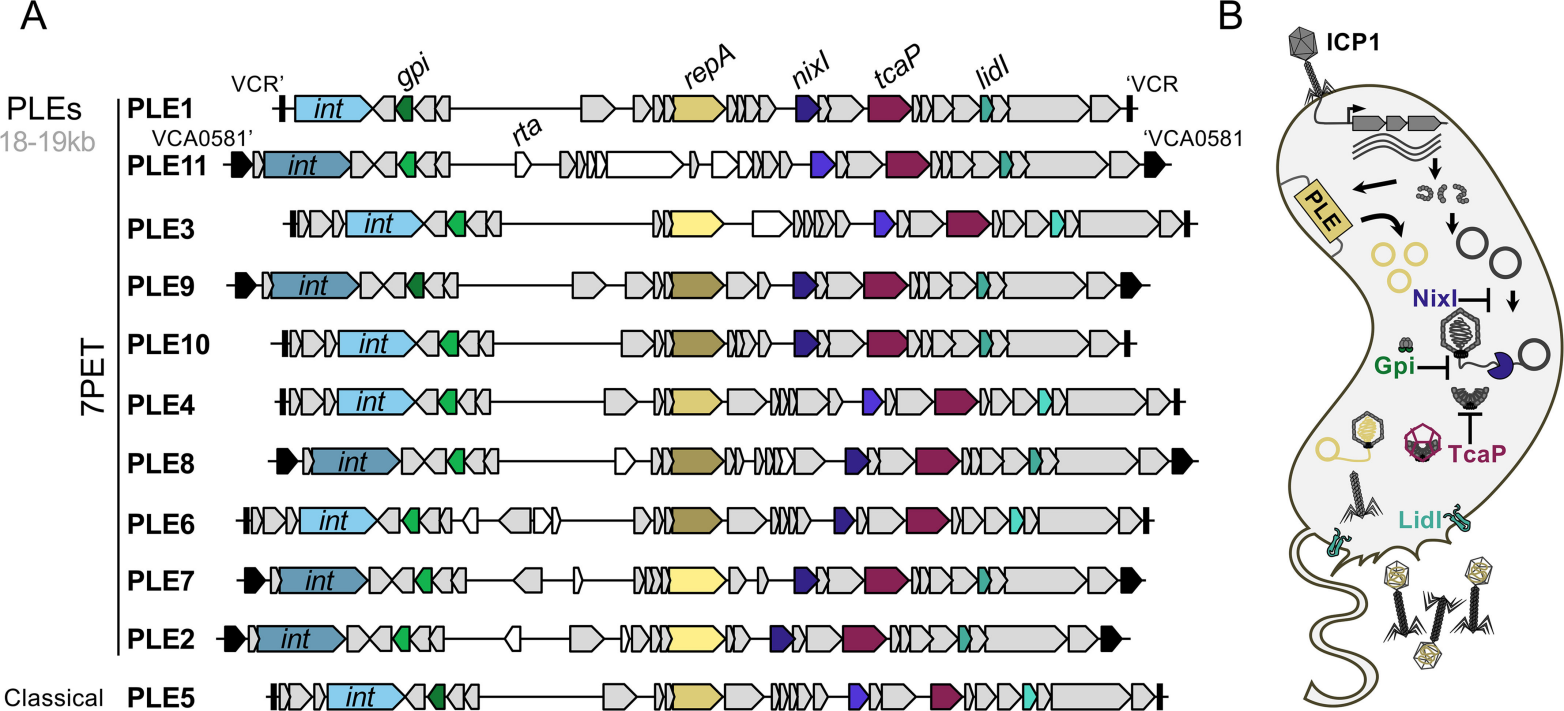
PLEs in 7PET and classical *V. cholerae*. (A) Genomic organization of PLEs. Shades of the same colour represent clusters of alleles grouped by amino acid similarity. Genes in white are unique to specific PLE variants (<30% amino acid identity to other PLE proteins). PLE insertion sites are marked by flanking genes/sites in black; VCR indicates the *V. cholerae* repeat in the superintegron. Key genes are indicated: the integrase (*int*) drives excision and integration, *repA* drives PLE replication, and conserved inhibitors Gpi, NixI, TcaP and LidI have roles in ICPI inhibition and PLE dissemination. PLE11 encodes a novel inhibitor, Rta, that blocks ICP1 irrespective of ICP1-encoded counter-defences. (B) Model of PLE activity: upon ICP1 infection, PLE excises, replicates and hijacks ICP1 structural proteins to package its genome into modified virions for horizontal transmission. Multiple inhibitors encoded by PLE work together to disrupt the phage lifecycle at various stages: NixI limits ICP1 replication, Gpi prevents genome packaging, TcaP interferes with the assembly of large capsids required to house the larger phage genome and LidI accelerates host cell lysis. Together, these activities abolish phage production and enable the release of PLE virions. These virions deliver the PLE genome into recipient cells, where it integrates into the chromosome (not shown).

Phage-inducible chromosomal island-like elements (PLEs) are a family of integrated mobile elements in *V. cholerae* that provide robust defence against ICP1 [47], the predominant phage in cholera patient stool samples. Ranging in size from 18.0 to 19.3 kb, PLEs encode approximately 26–31 predicted ORFs. While PLEs share genomic synteny and regions of nucleotide conservation, they have a mosaic architecture, with each PLE having a unique combination of the genes that encode the necessary products to carry out a conserved response to ICP1 infection (figure 2A). Notably, the gene products that execute these functions can differ significantly between PLEs [48], and additionally, some PLEs possess unique genes not found in other PLE variants.

To date, 11 PLE variants have been identified, having been detected in strains dating back to 1931 [48,49]. One PLE variant appears restricted to Classical Biotype strains (figure 2A), whereas the other 10 variants have been found in 7PET strains. While a comprehensive analysis of the distribution of PLEs among wave 1, 2 and 3 strains has not been done, PLEs are found in both wave 2 and wave 3 strains. Based on isolation dates of PLE6(+) and PLE7(+) strains (1987−1989) [48], PLEs may have also been present in wave 1 strains. Among 7PET strains, the distribution of PLEs is patchy and appears somewhat geographically constrained. For instance, wave 3 strains causing the ongoing outbreaks in Haiti and Yemen do not harbour PLEs. In contrast, in Bangladesh, PLE1 was prevalent in circulating *V. cholerae* strains of the BD-2 lineage between approximately 2009 and 2018 before being displaced by PLE(−) strains of the BD-1.2 sub-lineage of wave 3 strains in 2019 [48,50].

Experimental studies on PLE–ICP1 interactions have primarily focused on PLE1, the first variant discovered [51]. These studies have revealed that PLEs act as potent parasites of ICP1, exploiting phage resources to promote their own spread while simultaneously abolishing phage production. Upon ICP1 infection, PLE excises from the chromosome [52], replicates [53,54] and hijacks ICP1-encoded structural proteins to package its genome into virions for horizontal transmission. PLEs employ multiple mechanisms that act synergistically to interfere with the progression of ICP1’s lifecycle [55–58], thus protecting the *V. cholerae* population (figure 2B).

The first PLE-encoded protein to act to inhibit ICP1 upon infection is NixI, a sequence-specific nicking endonuclease that targets the phage’s genome during the transition from bidirectional theta to rolling circle replication [56]. By disrupting rolling circle replication—a process essential for generating linear genome concatemers that serve as the substrate for genome packaging—NixI significantly restricts the production of phage progeny (figure 2B). Concurrently, PLE’s TcaP, an external scaffolding protein, alters capsid assembly by redirecting ICP1’s coat proteins to form small procapsids unable to accommodate the full length of ICP1’s approximately 125 kb genome [57] (figure 2B). Following replication, PLE employs Gpi to target ICP1’s large terminase (TerL), the motor protein necessary for packaging the genome into procapsids, effectively blocking this step [58]. Finally, LidI, a PLE-encoded membrane protein, disrupts lysis inhibition, interfering with ICP1’s strategy to delay cell lysis and increase progeny production under high phage density conditions [55] (figure 2B). Each of these PLE-encoded proteins independently inhibits ICP1 production to varying degrees. However, the combined activity of these inhibitors (and possibly more) completely abolishes phage production and likely limits ICP1’s evolutionary escape through modification of PLE-targeted components.

ICP1 has evolved multiple anti-PLE mechanisms to counteract PLE’s ability to hijack its lifecycle and restore phage production. These counter-defence strategies vary across phage isolates [45] and target the PLE genome for nucleolytic degradation via distinct mechanisms. One such mechanism involves ICP1’s attachment-directed inhibitor Adi, which exploits the PLE-encoded integrase to induce destructive nuclease activity at the PLE circularization junction generated during excision [59]. Another, the origin-directed nuclease Odn, cleaves the PLE origin of replication [60]. However, PLEs encode diverse integrases and replication modules, some of which evade these phage-encoded counter-defences. As a result, Adi and Odn exhibit narrow spectrums of activity, antagonizing only specific subsets of PLE variants [48,49,59,60]. Therefore, this molecular arms race between ICP1’s counter-defences and PLE resistance mechanisms likely drives the modularity and diversification of PLEs.

Notably, ICP1’s most robust adaptation in the arms race against PLEs appears to be its acquisition of a CRISPR–Cas system [51], which provides broad-spectrum counter-defence that can adapt to sequence variation in the PLE genome. However, a recently identified PLE variant, PLE11—isolated from wave 3 strains in Bangladesh in 2021—represents a notable exception [49]. Despite CRISPR-mediated nucleolytic degradation, PLE11 retains its capacity to block ICP1 production. This resistance is attributed to a novel anti-ICP1 factor called Rta, which disrupts phage tail assembly and neutralizes ICP1 independently of the integrity of the PLE genome.

Despite the progress in understanding PLE–ICP1 interactions, several questions about the evolution and distribution of PLEs remain unresolved. One intriguing observation is that PLE variants tend to disappear as new ones emerge [47,48], suggesting that newly evolved variants may have a fitness advantage over their predecessors. However, determining the nature of such an advantage is challenging due to limited knowledge of the ICP1 genotypes prevalent during historical sampling. Moving forward, systematic efforts to survey and characterize phages from cholera patient stool samples may provide insights into these epidemiological patterns and their underlying mechanisms.

Another interesting observation is the absence of natural *V. cholerae* strains harbouring more than one PLE [47,48,61]. The lack of PLE co-occurrence within a single bacterial genome is unique among all families of phage parasites [61] and cannot be attributed to limited integration sites. Most PLEs integrate into a sequence referred to as the *V. cholerae* repeat [47,48,52], a sequence found over 100 times in the superintegron (figures 1 and 2A). The patchy distribution of PLEs among 7PET strains, coupled with their lack of co-occurrence within a single genome, may suggest that PLEs impose a fitness cost on *V. cholerae*. Consequently, the selective advantage of PLE-mediated defence against ICP1 might depend on the ecological context, with PLE(−) strains potentially out-competing their PLE(+) counterparts under certain conditions.

## 4. Sulfamethoxazole and trimethoprim-type integrative and conjugative elements (SXT ICEs)

**Figure 3.**
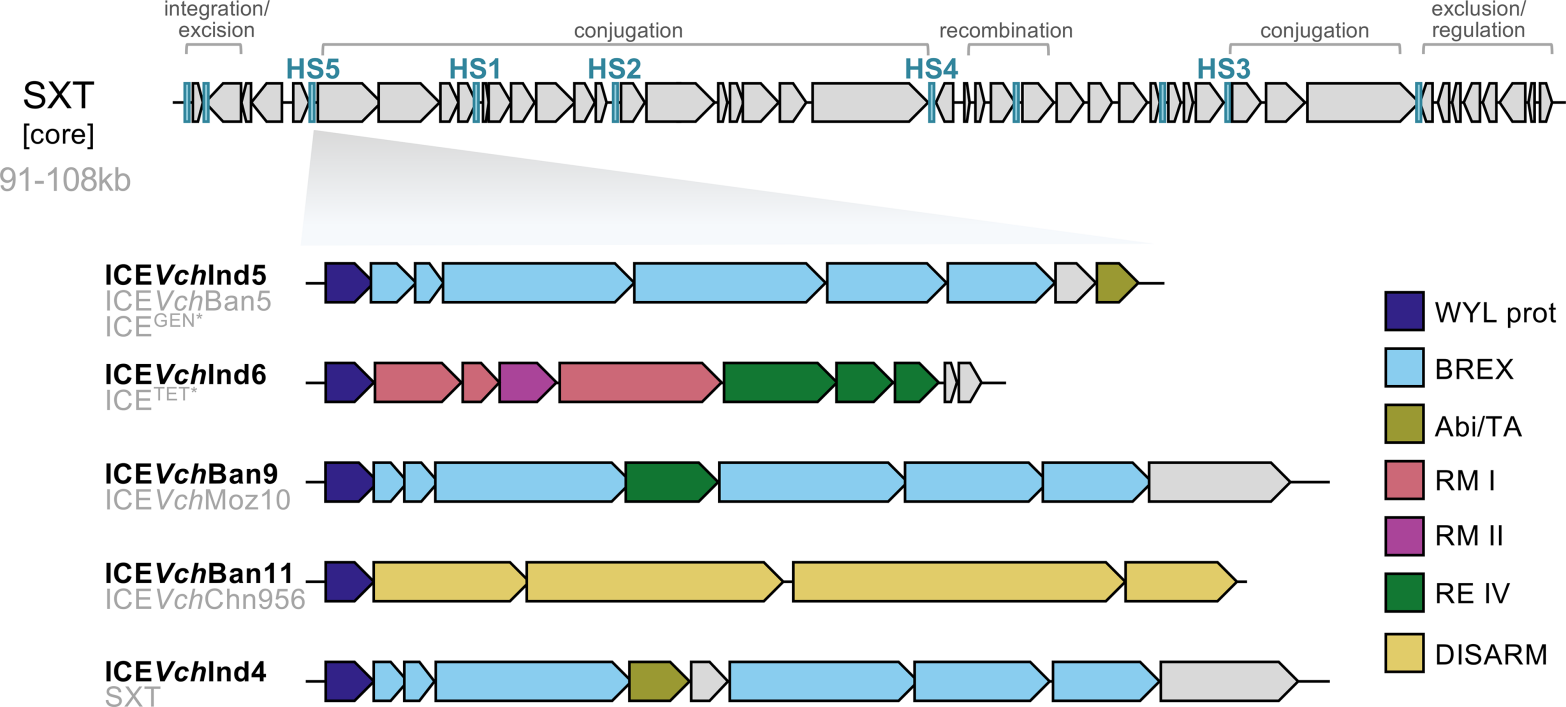
SXT ICEs in the wave 2 and 3 lineages of 7PET. Genomic organization of SXT ICE core genes (modified from Ryal *et al.* [63]) with core functions labelled above the schematic. Insertion of variable cargo DNA (blue lines) with hotspot regions (HS) numbered. All SXT ICEs encode anti-phage systems in hotspot 5. (Below) Expanded view of anti-phage systems in elements from 7PET strains, with primary names in black and other commonly used names in grey. Elements are arranged from top to bottom in order of decreasing prevalence in 7PET strains [62]. The size of SXT ICEs corresponds to the range of sizes for the five elements found in 7PET strains. ICE*Vch*Ind4 is 95% nucleotide identical to the original SXT discovered in MO10 but lacks *dfr18* [16].

ICEs of the SXT/R391 family are recognized as key drivers of the spread of antibiotic resistance in 7PET *V. cholerae*. Decades after their discovery, their role in phage defence was uncovered [62], illustrating how these MGEs promote adaptation to diverse environmental challenges. SXT ICEs are self-transmissible by conjugation, enabling dissemination beyond *V. cholerae* to a wide range of Gammaproteobacteria [63]. These elements are characterized by a syntenic and conserved set of core genes responsible for their integration/excision, conjugation and regulation, which acts as a scaffold for variable cargo genes [64] (figure 3). These adaptive cargo genes cluster in five intergenic hotspots [16], facilitating frequent inter-ICE homologous recombination [65]. The first identified SXT ICE was discovered for its antibiotic resistance genes for sulfamethoxazole and trimethoprim (abbreviated as SXT), giving this family of ICEs its name [17].

The first SXT ICE, ICE*Vch*Ind4, was identified in a *V. cholerae* O139 strain isolated in India in 1992 [17]. This marked the transition from wave 1 to wave 2 of the 7PET pandemic lineage [2], as wave 1 strains lack SXT ICEs, whereas wave 2 and wave 3 strains variably harbour one of five SXT ICEs: ICE*Vch*Ind5, ICE*Vch*Ind6, ICE*Vch*Ban9, ICE*Vch*Ban11 and ICE*Vch*Ind4 [62,65] (figure 3). Traditional nomenclature for SXT ICEs reflects the host species, geographic origin and a distinguishing number (e.g. *Vch*Ind5 for *V. cholerae* India 5) [66]. However, inconsistencies arise as identical SXT ICEs are found in different hosts and geographic regions, resulting in a lack of unification that can be confusing.

The role of SXT ICEs in phage defence was uncovered through clinical surveillance of *V. cholerae* and ICP1 in Bangladesh [62]. *Vibrio cholerae* isolates were susceptible to contemporary ICP1 phages but restricted phages from past or future time points, indicating the presence of a temporally fluctuating resistance determinant. This resistance was ultimately attributed to ICE*Vch*Ind5 and ICE*Vch*Ind6, whose frequencies varied during the sampling period. Further analysis revealed that all SXT ICEs encode phage defence systems within a single hotspot (figure 3). Among the SXT ICEs in 7PET strains, ICE*Vch*Ind5, ICE*Vch*Ban9 and the first discovered ICE*Vch*Ind4 carry bacteriophage exclusion (BREX) systems, which restrict phages through an epigenetic modification-dependent mechanism that remains poorly understood [67]. ICE*Vch*Ind6 encodes multiple restriction-modification systems, while ICE*Vch*Ban11 features a DISARM (Defence Island System Associated with Restriction-Modification) system [68]. Additional genes of unknown function are also present in this hotspot and may contribute to phage defence.

SXT ICE-encoded defence systems provide broad-spectrum protection against diverse phages [62], contrasting with the specificity of PLE-mediated defence discussed earlier. Experimentally, ICE*Vch*Ind5, ICE*Vch*Ind6 and ICE*Vch*Ban9 were shown to individually confer resistance to one or more of the ICP phages. Moreover, when introduced into *Escherichia coli*, these ICEs conferred varying degrees of resistance to unrelated phages, underscoring their broad defensive activity [62]. ICE*Vch*Ind4 and ICE*Vch*Ban11, which are rare among 7PET strains, have not, to our knowledge, been assessed experimentally for phage defence.

Similar to what is seen with PLEs, the dominant phage ICP1 coevolves with 7PET strains to counteract SXT ICE-mediated defences. For example, ICP1 counters ICE*Vch*Ind5 through the activity of the anti-BREX protein OrbA [62,69]. It can also escape ICE*Vch*Ind6 via stochastic epigenetic modifications. Another noteworthy parallel to what is observed with PLEs is that ICP1 infection can trigger SXT ICE conjugation, promoting the horizontal transfer of both phage defence and antibiotic resistance genes [62]—a potential complication for the application of phage therapy.

The clinical relevance of SXT ICE-mediated phage defence was recently highlighted by metagenomic analysis of cholera patient stool samples, which revealed a correlation between the presence of ICE*Vch*Ind5 or ICE*Vch*Ind6 and lower phage-to-*V. cholerae* ratios [46]. Under laboratory conditions, ICE*Vch*Ind5 abolishes ICP1 Δ*orbA* plaque formation entirely, and no escape mutants have been identified [62]. Even when ICP1 expresses OrbA as a counter-defence, ICE*Vch*Ind5 modestly restricts ICP1, reducing plaquing efficiency fourfold compared to strains lacking the phage defence hotspot [69]. However, while metagenomic analyses indicated that ICE*Vch*Ind5-mediated protection against ICP1 was not absolute [46], the genotype of the co-occurring ICP1 in patient samples was not examined. Consequently, it remains unclear whether ICE*Vch*Ind5 provides significant phage defence during human infection independent of phage-encoded counter-defence or if its protective effect is mitigated by specific ICP1 adaptations, as might be expected.

The appearance of SXT ICEs in the approximately 1980s reduced the effectiveness of clinically relevant antibiotics for cholera treatment and significantly shaped the spread of 7PET *V. cholerae* [2]. Although their well-documented role in antibiotic resistance has been widely regarded as the primary driver of their persistence in pandemic strains, the ability of SXT ICEs to mediate phage defence likely plays an equally critical role in ensuring the success and prevalence of 7PET lineages.

## 5. West African–South American-1 (WASA-1) prophage

**Figure 4.**
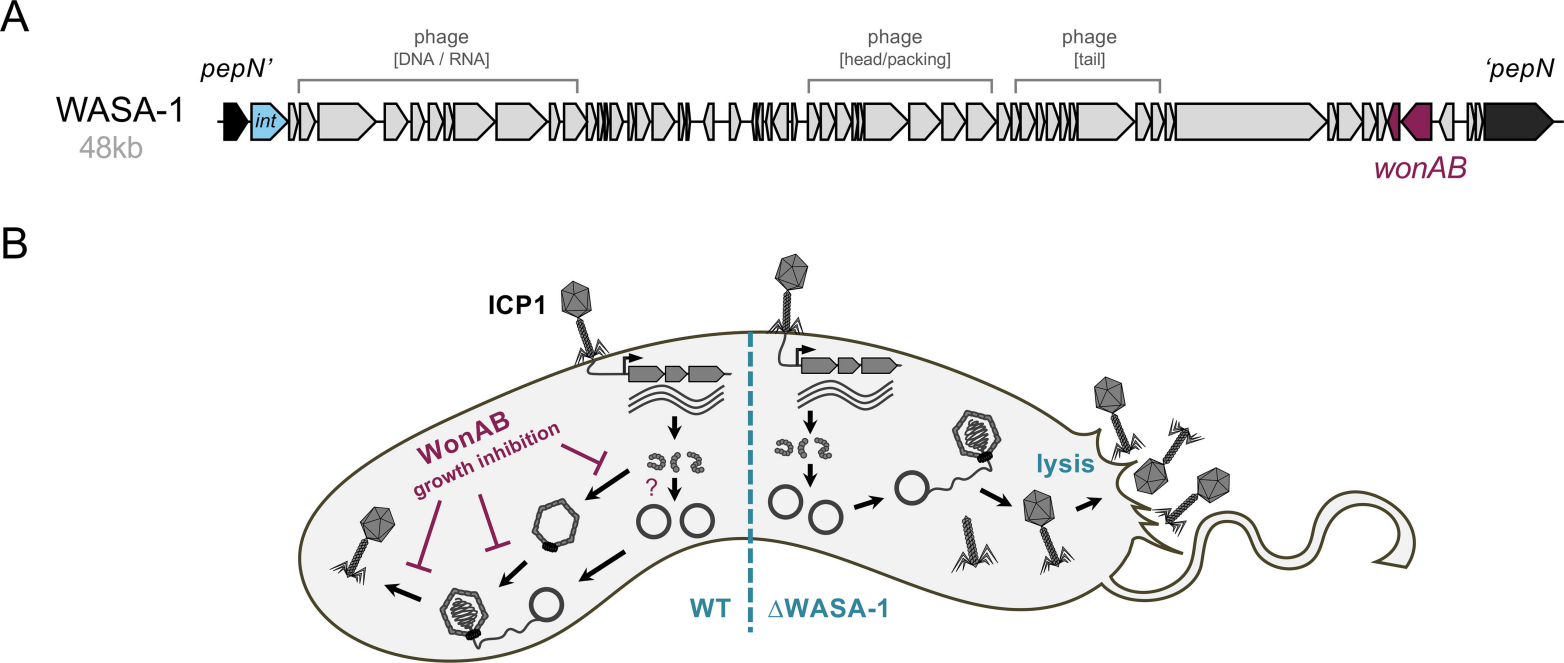
WonAB defence system in the wave 1 WASA lineage. (A) Schematic representation of the WASA-1 prophage region, with the *wonAB* defence system genes highlighted. (B) Illustration of WonAB-mediated protection against ICP1 infection. Wild-type (WT) strains with the WASA-1 element exhibit resistance to ICP1 infection (left), while strains lacking the WASA-1 element show complete lysis (right). WonAB-mediated growth inhibition aborts the infection process, resulting in no or only negligible capsid assembly and no detectable production of new infectious particles. A minor effect on DNA replication might also occur (indicated with ‘?’). ICP1 infection details are as described in figure 2.

While the research outlined above provided strong evidence for the arms race between ICP1 and MGEs, a key question remained: how did wave 1 strains of 7PET *V. cholerae* defend themselves against ICP1? These strains lack SXT and are thought to either not carry PLE or only carry it infrequently as an ICP1-defensive element. In this context, recent research by the Blokesch lab demonstrated that the WASA lineage of wave 1 strains, including extensively studied isolates such as A1552, C6706 and C6709, exhibits complete protection against a broad range of ICP1 phages [19], including recent isolates from the Democratic Republic of the Congo [70]. This protection is mediated by the 48 kb WASA-1 prophage [18,71], a hallmark of the WASA lineage [2,4] (figure 4A). Notably, while this element contains conserved phage-related genes, its status as an active phage remains undetermined at present.

Upon further investigation of the WASA-1 prophage, followed by systematic gene deletions targeting those genes or operons constitutively expressed under laboratory conditions, a two-gene operon responsible for ICP1 protection was identified and named *wonAB* [19] (figure 4A). The *wonAB* operon was shown to be both necessary and sufficient for this protective phenotype, as demonstrated by operon deletion and the expression of *wonAB* in non-WASA strains of *V. cholerae*, respectively [19]. Moreover, when expressed in *E. coli*, WonAB exhibited activity against all members of the *Vequintavirinae* subfamily of coliphages, suggesting that its protective function extends beyond ICP1 [19].

By tracking bacterial growth and division post-infection through absorbance measurements and microscopy, it was revealed that the WonAB system protects against ICP1 via growth inhibition (figure 4B) [19], a form of abortive infection [72]. Consistent with this finding, ICP1-infected wild-type WASA strains rarely contained assembled capsids and produced no detectable infectious particles (figure 4B). Moreover, attempts to isolate ICP1 phage escape mutants against WonAB were unsuccessful [19]. This observation suggests that WonAB may detect phage DNA or host DNA modifications in a way that ICP1 cannot evade. Further studies are underway to elucidate the precise mechanisms involved in the recognition of phage infection.

Bioinformatic analysis revealed that the sensor protein WonA belongs to the ABC-ATPase clade of ATPases and contains ATPase motifs characteristic of the overcoming lysogenization defect (OLD) ABC-ATPase family [19]. The effector WonB is predicted to possess a variant of the PD-(D/E)xK nuclease fold. Based on the conservation of these motifs, the system resembles a subclass named OLD-ABC ATPase+Novel REase, which was described in a recent *in silico* classification of ABC ATPases by Krishnan *et al.* [73]. Consequently, the system was named WASA OLD-ABC ATPase Nuclease (WonAB) [19]. Homologs of WonAB have been identified across various Gram-negative bacteria [19].

Based on these data, it is tempting to speculate that the WASA-1 prophage, combined with a VSP-II variant encoding two novel phage defence systems [19], played a direct role in the West Africa–South America transmission event in the 1990s. It is worth noting that despite a century of cholera absence, Peru experienced one of the most severe multi-year epidemics in history between 1991 and 1997 caused by the WASA lineage of 7PET [74]. During this period, over a million cholera cases were reported from 1991−1993 alone, resulting in nearly 9000 fatalities attributed to the disease [74].

## 6. Defence systems in classical *Vibrio cholerae* strains

The classical lineage of *V. cholerae* is known to have caused the fifth and sixth cholera pandemics and likely earlier pandemics as well [75]. Differentiation between classical and 7PET strains is often based on phenotypic differences, such as hemolysis and enhanced resistance to polymyxin B in 7PET strains, the latter of which is conferred by the addition of glycine to surface-exposed LPS via the AlmEFG proteins [76]. Notably, in classical strain isolates such as O395, 569B and A103, *almF* carries a 49 bp deletion causing a frameshift, which explains their sensitivity to polymyxin B. Recent comparative genomic analyses have shown that the classical and 7PET lineages independently evolved from a common ancestor [1,2]. It is, therefore, not surprising that they exhibit significant differences in their sensitivity to various phages, which underpin the phage typing schemes described above. Investigating the defence systems of classical strains is thus an important and active area of research, as outlined below.

### (a) CRISPR-Cas system

**Figure 5.**
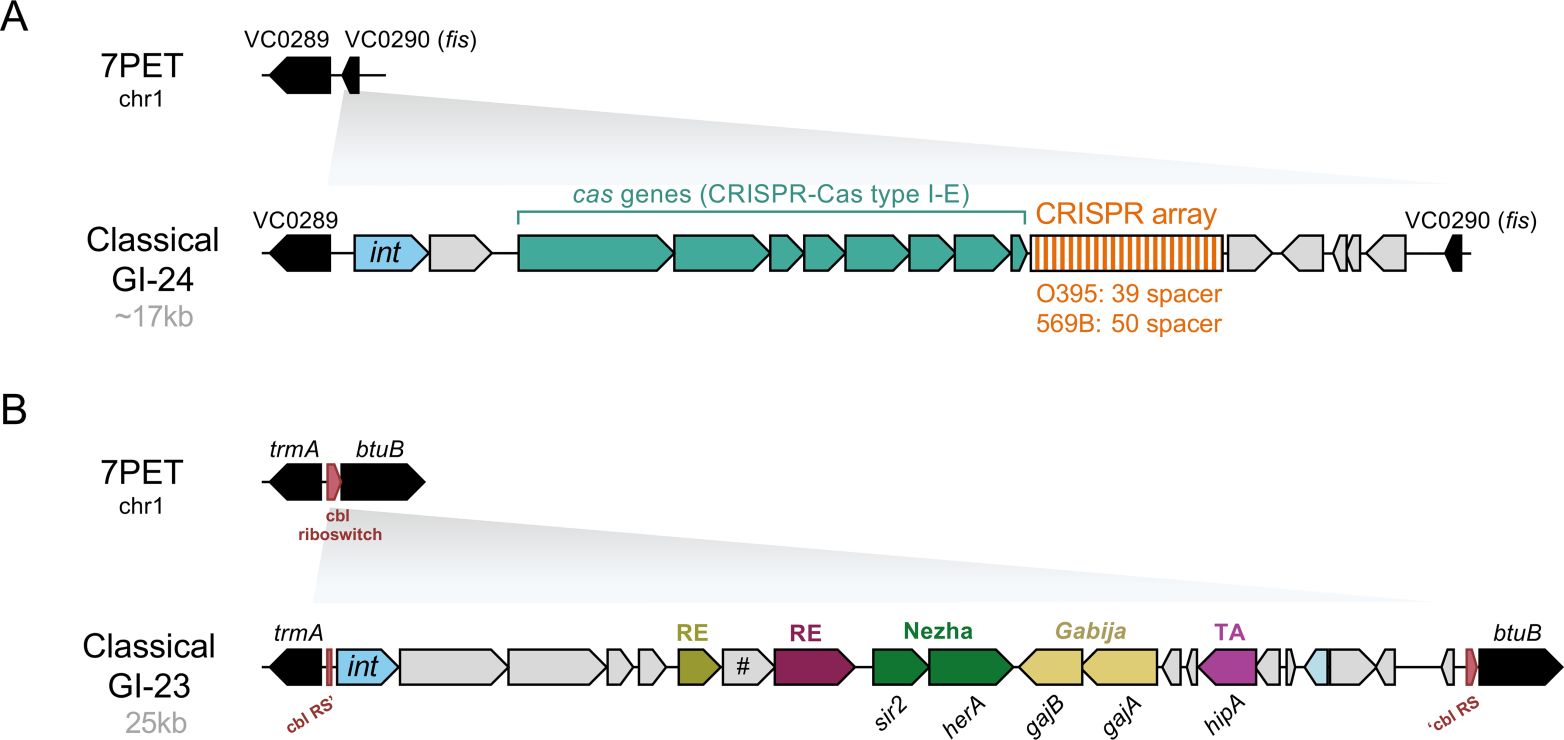
MGEs and defence systems in classical *V. cholerae*. (A, B) Schematic of GI-24 and GI-23 genomic islands in classical *V. cholerae*, encoding CRISPR–Cas or the Nezha and Gabija defence systems, respectively. Key elements include the cobalamin riboswitch (Cbl RS), toxin–antitoxin module (TA) and predicted restriction endonucleases (RE). The figure also highlights a frameshifted *IS3* family transposase gene (#).

Since the discovery of CRISPR–Cas systems (recently reviewed by Ishino *et al.* [77]), it has been shown that many bacterial and archaeal species possess these systems, with prediction tools now readily available [78–80]. The presence of CRISPR–Cas systems in *V. cholerae* is also frequently mentioned in the literature. However, in this context, it is essential to differentiate between the various *V. cholerae* lineages and strains. To date, no 7PET strain encoding CRISPR–Cas systems on its two primary chromosomes has been identified. Instead, CRISPR–Cas systems are commonly found in members of the classical lineage [1,81,82] (figure 5A) and in non-pathogenic and/or environmental isolates of *V. cholerae* [83–85]. The same applies to CRISPR-associated transposons (CASTs) [86], including the well-characterized *Vc*CAST transposon (Tn*6677*; [84,87]) identified in the environmental non-O1/non-O139 Haitian isolate HE-45. Notably, this isolate also harbours a hybrid CRISPR–Cas type III-B/I-F system, which has been reported to be associated with a prophage [84].

In classical strains, CRISPR–Cas systems were initially identified within the 17 kb genomic island GI-24 [1] (figure 1), which integrates downstream of the *VC0290* gene on the large chromosome (figures 2 and 5A). This gene encodes the nucleoid-associated protein factor for inversion stimulation (Fis). The CRISPR–Cas system in classical strains belongs to the type I-E category, distinguishing it from the type I-F CRISPR–Cas systems frequently found in ICP1, which function as counter-defence mechanisms against PLEs, as described above [51]. Moreover, the spacer diversity observed in classical strains’ type I-E system supports its role in phage defence, a functionality that has been experimentally demonstrated [81].

### (b) Nezha defence system protects from ICP phages

Recent work by the Camilli lab identified two new defence systems in sixth pandemic classical strains that are responsible for the strains’ resistance against the virulent phages ICP1, ICP2 and ICP3. Briefly, Woldetsadik *et al.* identified a 25 kb genomic island in classical *V. cholerae* isolates, reportedly located between the genes *tonB* and *trmA* [88]. However, closer inspection revealed that this genomic island is inserted into the cobalamin riboswitch upstream of the vitamin B12 transporter gene *btuB*, with the correct flanking genes being *btuB* (VC0156) and *trmA* (VC0154) (figure 5B). This discrepancy likely stems from the initial annotation of the VC0156 gene as a ‘TonB-dependent receptor’ in the 2007 genome sequence of the classical strain, deposited under accession number NC_009457 in NCBI.

This genomic island, initially named GI-23 [1], is specific to classical strains and encodes several predicted or putative anti-phage systems [88] (figure 5B). Among these, the authors focused on two systems, Nezha and Gabija, identifying Nezha as the dominant factor responsible for the observed resistance against ICP phages [88]. Of note, ICP2 infections rely on the outer membrane porin OmpU as a receptor [40,89]. Previous studies demonstrated that the amino acids V324 and G325 are frequently mutated in patient isolates from Bangladesh and Haiti, impairing ICP2 infection [40,89]. Interestingly, the classical strain A103 tested by Woldetsadik *et al*. [88] carried OmpU variants with V324A and G325S, as do other classical isolates such as the commonly studied strains O395 and 569B, rendering them similarly resistant to the ICP2 isolates tested in their study. Consequently, defence system-mediated protection against ICP2 could only be evaluated in receptor-reconstituted variants of the strain, in which valine and glycine were restored at positions 324 and 325, respectively [88].

Nezha is a two-component defence system consisting of a Sir2 NADase and a HerA helicase [90,91]. Upon activation, this system depletes nicotinamide adenine dinucleotide (NAD^+^) within the cell, inhibiting phage replication. While the protective phenotype against ICP1 phages was very strong, the authors identified an ICP1 variant isolated in 2001 that could overcome the defence system. Using elegant phage recombineering and ICP1-CRISPR/Cas-based selection, a two-gene operon was identified in this phage that encodes NAD^+^ regeneration enzymes, named the NAD^+^ Regenerating System AB (*nrsA* and *nrsB*). These gene products function as adenosine diphosphate ribose-pyrophosphate synthetases (Adps) and nicotinamide ADPR-transferases (Namat), respectively. A similar anti-defence mechanism was concurrently described by Osterman *et al.* for other organisms [92]. Due to the enzymatic activity of NrsA and NrsB, the phage restores NAD^+^ levels in the host cell, countering the immunity effect of Nezha and allowing the phage to replicate and produce new infectious particles. Interestingly, the majority of ICP1 phages were shown to carry this operon, but the genes are often non-functional, likely due to the fitness cost imposed by NrsAB in cells that do not encode the Nezha system. Because sequenced 7PET strains mostly lack the Nezha system, this explains why most modern-day ICP1 isolates carry non-functional *nrsAB*. However, experiments demonstrated that the reversion of two mutations in *nrsB*—one a missense mutation and the other a nonsense mutation—could occur spontaneously, thereby restoring the system’s functionality and counter-defence against Nezha.

## 7. Discussion and future perspectives

As outlined above, pandemic *V. cholerae* strains from both the classical and 7PET lineages have evolved sophisticated defence systems to protect themselves against viral predators. These differences in their defence repertoire likely contributed to the phage typing patterns documented primarily during the twentieth century. Unfortunately, it is now challenging or even impossible to obtain these historical phages, which would be of great value for current research. Even when such older phages are available, there is a risk that they may have been inadvertently contaminated, rendering the original phages no longer viable. For instance, the authors of this work contacted numerous researchers and culture collections in an attempt to locate a reliable source for the 10 phages described by Basu & Mukerjee [30] and Chattopadhyay *et al*. [31]. Thus, future efforts to expand the collection of vibriophages capable of infecting *V. cholerae*, similar to the BASEL coliphage collection [93], would be greatly appreciated. Such a resource, if sampled on non-7PET *V. cholerae* or genetically engineered defence mutants, could particularly enhance our understanding of the defence systems encoded on the core MGEs—VPI-2, VSP-1 and VSP-II—of 7PET strains [26].

However, although not numerous, the phages that are widely shared with the research community are of immense value. Phages from the ICP1, ICP2 and ICP3 families [44], for instance, have been instrumental in uncovering lineage- or even strain-specific defence mechanisms in *V. cholerae*. These include the identification of the PLE, the SXT phage defence hotspots, the WASA-1-encoded WonAB system and the Nezha and Gabija systems in classical *V. cholerae*, as outlined above. Furthermore, these phages have played a crucial role in uncovering rare defence systems in 7PET *V. cholerae*. For example, a DarTG defence system was recently identified in three clinical *V. cholerae* isolates from 2008 to 2009, which was shown to inhibit ICP1 replication [94]. The DarTG toxin–antitoxin system comprises an ADP-ribosylase, DarT and a de-ADP-ribosylase, DarG, which functions as the antitoxin. Following an unknown trigger after phage infection, DarT ADP ribosylates the phage DNA, inhibiting phage replication and RNA synthesis, thereby aborting the phage infection [95]. ICP1 isolates can encode an anti-DarT antitoxin (anti-DarT factor B, AdfB) to circumvent the DarTG system [94], suggesting that even seemingly rare phage defence systems in 7PET *V. cholerae* are met by counter-adaptations in prevalent phages.

ICP phages have also been instrumental in uncovering a phage defence element, originally described as GI-23 [1], within the classical lineage. This element encodes several defence systems, including Nezha and Gabija [88], yet studies on defence mechanisms in classical strains remain limited. An exception to this is the past research on CRISPR–Cas systems. In this context, a long-standing mystery in the field remains: why 7PET strains never encode CRISPR–Cas systems, despite their prevalence in classical strains and environmental *V. cholerae*. Notably, Box *et al.* demonstrated that transferring the classical CRISPR–Cas system into a 7PET strain via natural transformation allows it to function effectively in the new host [81]. This observation raises the intriguing possibility of mutual exclusion between the universal defence systems present in 7PET strains [26] and CRISPR–Cas systems. Further research will be needed to determine whether the presence of functional CRISPR–Cas systems imposes fitness defects in 7PET strains, which could explain their absence in this lineage.

CRISPR spacer sequences matching vibriophages used in historical phage typing schemes have been identified [81]. For instance, phage X29 [29,41,96] is frequently targeted by CRISPR–Cas in classical and other non-PET *V. cholerae* [81,84], suggesting that these strains often encountered X29 or its close relatives. Interestingly, although 7PET strains lack CRISPR–Cas systems, they have evolved alternative defence mechanisms against phage X29. These mechanisms include the DdmABC system, encoded by VSP-II, and the *Vc*SduA system, which is encoded in a WASA-lineage-specific variant of VSP-II [19,97,98] (see accompanying article by Blokesch [26]).

Lastly, the sampling of 7PET *V. cholerae* and ICP phages over time, with a particular focus on ICP1, has provided a rare opportunity to examine the flux of defence systems as the pathogen evolves. This includes the acquisition, loss, mutation and mutation reversion of anti-defence systems in the phage. Indeed, studies on the coevolution of *V. cholerae* and ICP1 represent one of the few systems available that enable the study of phage defence in a native context, spanning from patient samples down to the molecular level.

Collectively, the findings reported in this review highlight the complex interplay between phage defence systems and the evolutionary pressures faced by *V. cholerae* across different lineages. These observations underscore the need for further research to unravel the molecular mechanisms driving these adaptations and to continue and expand efforts to monitor the ongoing arms race between 7PET *V. cholerae* and its predatory phages.

## Data Availability

This article has no additional data.
